# The Microbiological Spectrum and Antibiotic Resistance in Acute Acalculous and Calculous Cholecystitis: A Seven-Year Study in a Tertiary Center

**DOI:** 10.3390/medicina61112028

**Published:** 2025-11-13

**Authors:** Cosmin Vasile Obleaga, Ovidiu Mircea Zlatian, Oana Mariana Cristea, Alexandra Rosu-Pires, Alexandru Marin Pascu, Mirela-Marinela Florescu, Claudiu Marinel Ionele, Ion Rogoveanu, Alexandru Valentin Popescu, Vlad Catanoiu, Sergiu Marian Cazacu

**Affiliations:** 1 Surgery Department, University of Medicine and Pharmacy Craiova, Clinical Emergency Hospital Craiova, 200642 Craiova, Romania; cosmin.obleaga@umfcv.ro; 2 Microbiology Department, University of Medicine and Pharmacy Craiova, Clinical Emergency Hospital Craiova, 200642 Craiova, Romania; oana.cristea@umfcv.ro; 3 Gastroenterology Department, University of Medicine and Pharmacy Craiova, Unidade Local de Saúde de Almada-Seixal, 2805-267 Almada, Portugal; alexandra.rosu@umfcv.ro; 4 Surgery Department, Clinical Emergency Clinical Hospital Craiova, 200642 Craiova, Romania; pascu.alexandru07@gmail.com; 5 Pathology Department, University of Medicine and Pharmacy Craiova, Clinical Emergency Hospital Craiova, 200642 Craiova, Romania; mirela.florescu@umfcv.ro; 6 Gastroenterology Department, University of Medicine and Pharmacy Craiova, Clinical Emergency Hospital Craiova, 200642 Craiova, Romania; claudiu.ionele@umfcv.ro (C.M.I.); ion.rogoveanu@umfcv.ro (I.R.); sergiu.cazacu@umfcv.ro (S.M.C.); 7 Gastroenterology Department, Doctoral School, University of Medicine and Pharmacy Craiova, Clinical Emergency Hospital Craiova, 200642 Craiova, Romania; popescualexandruvalentin97@yahoo.com; 8 Gastroenterology Department, Clinical Emergency Clinical Hospital Craiova, 200642 Craiova, Romania; vladcatanoiu@yahoo.com

**Keywords:** acute acalculous cholecystitis, acute calculous cholecystitis, bile culture, antibiotic resistance

## Abstract

*Background and Objectives*: Acute acalculous cholecystitis (AAC) is rare, mostly in older males, with cardiovascular diseases, diabetes, critical illness, or systemic infection. Antibiotherapy before or after cholecystectomy is important for preventing septic shock and postoperative infections. Increasing antibiotic resistance was recently noted and can complicate antibiotherapy. *Materials and Methods*: A retrospective study of all patients who underwent cholecystectomy between 2018 and 2024 in the Clinical Emergency Hospital of Craiova was performed. The etiology of AAC, complications, hospitalization duration, mortality, positive bile cultures, and in vitro antibiotic resistance were analyzed. *Results*: A total of 802 calculous and 54 AAC were recorded. Patients with AAC were predominantly males (OR = 1.767, *p* = 0.043) with diabetes (OR = 2.049, *p* = 0.014) and were older (66.6 ± 13.2 vs. 61.4 ± 15.6, *p* = 0.014). Mortality was significantly higher in AAC (18.5 vs. 3.6%, OR = 6.058, *p* < 0.001), with longer hospitalization (mean 9.7 vs. 8.4 days) and more perforation. Positive bile cultures were recorded in 60.5–66.2% of cases, with a similar etiology in both forms of acute cholecystitis (mostly Gram-negative species, *Enterococcus,* and *Staphylococcus*); 10 ESBL *Escherichia coli* and *Klebsiella* strains, 11 *Staphylococcus aureus* MRSA, and 1 *Enterococcus* VRE strain were recorded. Antibiotic susceptibility in vitro was similar in both AAC and calculous cholecystitis. Significant resistance to cephalosporins and quinolones was recorded; the lowest resistance was noted for amikacin, carbapenems, chloramphenicol, colistin (Gram-negative bacteria), and vancomycin. *Conclusions*: AAC was encountered in older males with diabetes, with a higher rate of complications and in-hospital mortality. Bile cultures were positive in 60.5–66.2%, predominantly with Gram-negative, *Enterococcus*, and *Staphylococcus* species. Significant in vitro resistance to cephalosporins and quinolones was found.

## 1. Introduction

Acalculous acute cholecystitis (AAC) represents a rare entity in adults (up to 5–15%), being more frequent in children (30–50%) [1,2,3,4,5,6,7,8,9]. Most patients are males and older than in calculous cholecystitis [3,5,10]; diabetes, atherosclerotic disease, and hypertension are frequently cited as risk factors for AAC [9]. The etiology includes dehydration, prolonged parenteral nutrition, end-stage renal disease, metabolic or cardiovascular diseases, severe diseases such as burns, trauma, neoplasia, cardiopulmonary resuscitation or mechanical ventilation, postoperative or post-transplantation, and systemic bacterial (Gram-negative or anaerobe) or viral infections [2,3,4,5]; some cases of acute acalculous cholecystitis were also described in severe immunosuppression, AIDS (Acquired Immunodeficiency Syndrome), systemic lupus, and vasculitis [1,3,5]. Although traditionally acute acalculous cholecystitis was described in critically ill patients, a recent study emphasized that 77 to 90% of patients were outpatient cases [5]. Cases associated with bacterial infections may include *E. coli*, *Enterococcus*, *Brucella* [2,8], *Leptospira* [10], or *Pneumococcus* [2], whereas viral infections such as Epstein–Barr virus, cytomegalovirus, varicella-zoster virus, or hepatitis A and C virus have also been noted [9,11,12]. Sepsis, meningitis, or hemolytic uremic syndrome may be associated with acute acalculous cholecystitis [2].

Several mechanisms are involved in acute acalculous cholecystitis; stasis, hypomotility, changes in bile pigment, and sepsis, together with ischemia and inflammation, can lead to increased luminal pressure and up to 10% perforation risk [3,4,5,6,7,9,13,14]. Direct invasion has been postulated for some agents, such as *Salmonella*, hepatitis A, Epstein–Barr, and COVID-19 infection and also for other infectious agents in immunocompromised hosts [9], while vasculitis, microangiopathy, or extensive thrombosis were incriminated in leptospirosis-, Zika virus-, *Rickettsia-*, or COVID-19-associated AAC [9,15]. Mechanical ventilation, concomitant sepsis, and total parenteral nutrition may have a significant role in severe cases of COVID-19 infection [15,16,17].

Clinical manifestations may be typical of acute calculous cholecystitis or may be covert in critically ill patients; jaundice is more frequent in sepsis-associated acute acalculous cholecystitis [3,18]. Transabdominal ultrasound showed a thickened gallbladder wall above 3.5 mm, a distended gallbladder with more than 8 cm in longitudinal diameter and 5 cm in transversal diameter, pericholecystic fluid, subserosa edema, intramural wall, and gallbladder sludge without stones [2,3,7,14].

The management of acute acalculous cholecystitis is based on antibiotherapy efficient for Gram-negative, anaerobes, and *Enterococcus*, analgesics, fluid replacement, and parenteral nutrition; cholecystectomy is usually indicated in cases complicated with gangrene or perforation [2], and cholecystostomy [6,19] or new approaches such as endoscopic-guided drainage (by ERCP or EUS) may be useful in selected cases, especially in critically ill patients [3]. The mortality is usually higher than in calculous cholecystitis because of the concomitant diseases, it being related to diagnostic delay, and the significant comorbidities [2,3,6]. A 10–30% average mortality was traditionally reported [2,7,12]. The recurrence rate after non-surgical treatment ranges between 2.7 and 22% [6,10].

Antibiotherapy in acute cholecystitis represents an important factor for decreasing mortality and complications, recommended in the first hour in patients with septic shock and within 6 h in other cases [1]. The role is prophylactic in early and non-severe forms, and curative in moderately severe or severe cases. Most bacteria involved are Gram-negative bacteria, followed by *Enterococcus*, while *Streptococcus*, *Staphylococcus*, and anaerobes are less frequently encountered [1]. The prevalence and susceptibility are influenced by several factors: community- or healthcare-associated type of infection and local susceptibility may play an important role [1]. We currently do not have enough regional data regarding microbiological patterns and susceptibility in acute cholecystitis; given local particularities of etiological factors and antibiotic susceptibility, differences regarding pathogenic mechanisms in acalculous and calculous acute cholecystitis and the lack of literature data regarding microbiological spectrum and antibiotic susceptibility in acute acalculous cholecystitis, a study detailing these aspects may help to choose empirical antibiotherapy in both acalculous and calculous acute cholecystitis.

The purpose of our study was to assess the prevalence and risk factors for acute acalculous cholecystitis following cholecystectomy, the microbiological pattern, and in vitro susceptibility to antibiotics in acalculous acute cholecystitis compared to calculous acute cholecystitis.

## 2. Materials and Methods

### 2.1. Patient Selection

We performed an observational, retrospective cohort study of patients with acute cholecystitis who underwent surgery between 1 January 2018 and 31 December 2024 in the Clinical Emergency Clinic Hospital of Craiova. Cases with acute cholecystitis but without surgery were excluded from the study, as bile cultures were unavailable. Data were collected from the analysis of the patient’s discharge documents from the Hippocrates computer system of the hospital (version 4, Romanian Software Solutions, Bucharest, Romania), with the procedure code of cholecystectomy (J101) and diagnostic codes of gallbladder stone with acute cholecystitis (K80.00), gallbladder stones without acute cholecystitis (K80.20), acute cholecystitis (K81), and peritonitis (K65). All pathological samples following cholecystectomies were analyzed; the cases showing pathological features of acute cholecystitis were included in the final analysis. The pathological diagnosis of acute cholecystitis was based on the presence of acute inflammation with neutrophils infiltrating the gallbladder wall, acute inflammatory processes (congestion, microabscess formation), and no significant fibrotic gallbladder wall thickening [20,21]. The diagnosis of chronic cholecystitis was based on the presence of fibrosis, Rokitansky–Aschoff sinuses, and chronic inflammatory infiltrate. Gangrenous cholecystitis was characterized by gallbladder wall necrosis associated with acute inflammation. Tokyo classification (Table 1) was employed for severity assessment [20,22,23,24], and Dindo–Clavien classification was used for complications assessment (Table 2).

### 2.2. Materials and Antimicrobial Susceptibility Testing

Bile sampling was performed by the surgical team in gangrenous and phlegmonous cholecystitis, in cases with purulent peritonitis (when both bile and peritoneal cultures were performed), and in cases with gallbladder hydrops; all included cases had intraoperative bile sampling. The samples were analyzed according to the clinical laboratory microbiological protocol; Gram-stained smears were performed, with immediate inoculation in blood broth media, incubation for 18 h at 37 °C, and then inoculation on blood agar, chocolate agar, and Drigalski and Sabouraud agar media in aerobic, anaerobic, and microaerophilic atmospheres obtained using the Genbag system (BioMérieux, Marcy-l’Étoile, France). Cases with positive culture were analyzed for antimicrobial resistance and evaluated by antimicrobial susceptibility testing; the presence of MDR (multidrug-resistant) and XDR (extensively drug-resistant) strains and the proportion of MRSA (methicillin-resistant *Staphylococcus aureus*), ESBL (extended-spectrum beta-lactamase), VRE (vancomycin-resistant *Enterococci*), or CPE (carbapenem-resistant *Enterobacterales)* were noted. The definition of MDR and XDR strains was based on the classification of the European Center for Disease Prevention and Control and the U.S. Centers for Disease Control and Prevention; MDR was defined as non-susceptibility to a minimum of one antibacterial agent from at least three antimicrobial categories, while XDR was defined as non-susceptibility to a minimum of one antibiotic in all but less than two antimicrobial categories [25]. The disk diffusion Kirby–Bauer method was used for antibiotic testing, according to the standard of the Clinical Laboratory Standards Institute (CLSI). Detection of ESBL and carbapenemase-producing *Enterobacteriales* (CPE) strains was made phenotypically [26] with the aid of the disk diffusion double-disk method from Rosco (Taastrup, Denmark), in which a 0.5 McFarland inoculum was placed on a Muller–Hinton agar plate together with five Rosco tablets (meropenem alone and in three inhibitor combinations plus temocillin) and incubated overnight. The zone-size differences were read according to the manufacturer’s instructions for the detection of CPE, MBL, AmpC-porin, or OXA-48 carbapenemases.

### 2.3. Study Approval

The study was approved by the Clinical Emergency Hospital of Craiova Ethics Committee (no. 14090/27.03.2025) on the following information: (1) data was collected within a retrospective, observational study; (2) the study did not interfere with current medical care; (3) data was collected and analyzed anonymously so that the patient data confidentiality would not be breached.

### 2.4. Statistical Analysis

The extracted data were tabulated in an Excel spreadsheet. Microsoft Excel 2019 MSO (version 2304 Build 16.0.16327.20200) was used in order to build a database with all the variables, and the MedCalc version 20.218 software was used for statistical analysis. We collected demographic data (age, gender), clinical symptoms and signs, evolution during admission and complications, the hospitalization period, laboratory analyses (hemogram, urea, creatinine, serum glucose and amylase, total and conjugated bilirubin, ALT, and AST), imaging (transabdominal ultrasound, CT scan, and MRI, if available), and pathology results. The frequencies were presented as absolute numbers and percentages. Chi-squared tests were used to compare ordinal or nominal variables. Continuous variables were compared using the Mann–Whitney U test if the variable was not normally distributed. A *p*-value smaller than 0.05 was considered statistically significant. Inverse probability weighting analysis was used to adjust proportions and mortality.

## 3. Results

### Characteristics of the Included Patients

**Figure 1:**
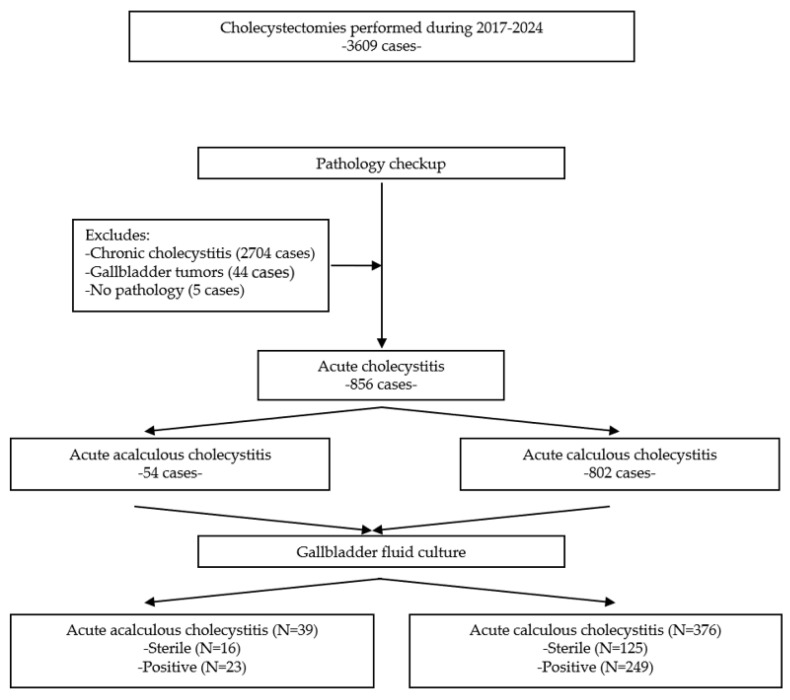
The flow chart for patients with acute acalculous and calculous cholecystitis.

From 1 January 2018 to 31 December 2024, a total of 856 cholecystectomies were performed for acute cholecystitis, of which 54 were acalculous and 802 were calculous (Figure 1). The mean age was higher in acalculous cholecystitis (66.6 vs. 61.4 years, *p* = 0.014); male predominance was noted in acute acalculous cholecystitis (57.4%) compared to female predominance (56.7%) in acute calculous cholecystitis (OR = 1.767, *p* = 0.043). The frequency of perforation and of phlegmonous and gangrenous forms was higher in acute acalculous cholecystitis, although statistical significance was not attained for gangrene. A total of 35.2% of acute acalculous cholecystitis patients had diabetes compared to 20.9% with acute calculous cholecystitis (OR = 2.049, *p* = 0.014); other comorbidities were similar. *Clostridioides difficile* colitis was noted slightly more frequently in acute acalculous forms (3.7 vs. 0.7%, *p* = 0.029), whereas the presence of COVID-19 infection was similar. Laboratory analyses have shown lower mean values of hemoglobin, lymphocytes, and platelet counts and slightly higher neutrophil counts in acute acalculous cholecystitis; significantly higher mean levels of urea and creatinine were also recorded in acute acalculous cholecystitis, which reflects the higher prevalence of acute renal injury, since the presence of chronic kidney disease was similar. Mean levels of aminotransferases and bilirubin were higher in acute calculous cholecystitis because of the presence of biliary obstruction. Complication occurrence (reflected by a higher Dindo–Clavien grade) was more frequent in acalculous forms, and hospitalization duration was longer. Mortality was much higher in acalculous forms (18.5 vs. 3.6%, *p* < 0.0001)—Table 3.

In 39 of 54 acalculous and 376 of 802 calculous acute cholecystitis, culture from the gallbladder fluid was performed, with 23 acalculous and 249 calculous cholecystitis having positive cultures (60.5 and 66.2%, respectively). All cases included in our study had intraoperatively bile sampling. Bile sampling was performed more frequently in acalculous compared to calculous cholecystitis (70.4 vs. 29.6%, *p* = 0.001). Patients with bile culture sampling were older (66.9% above 60 years vs. 54.8%, *p* = 0.004) and had open cholecystectomy more frequently (74.4 vs. 36.3%, *p* < 0.001), more severe forms (73.4 vs. 49.1% Tokyo II–III grades, *p* < 0.001), acute kidney injury more frequently (67.2 vs. 32.8%, *p* < 0.001), and a higher in-hospital mortality rate than those without bile culture (OR 3.841, 95% CI 1.3962–5.7859, *p* = 0.003). A higher leucocyte and neutrophil count (mean values 14,307 ± 6637 vs. 11,953 ± 6196, *p* < 0.001, and 11,578 ± 6347 vs. 9089 ± 6020, *p* < 0.001) and a lower lymphocyte count (1571 ± 823 vs. 1756 ± 950, *p* < 0.001) were also noted in patients with bile sampling; no differences were noted for ALT, AST, or bilirubin levels.

The etiology was similar, with a net predominance of Gram-negative species in both forms, followed by *Enterococcus* and *Staphylococcus * spp. *Serratia* being noted more frequently in acalculous forms (6.1% vs. 0.9%, *p* = 0.0391); *Enterococcus* prevalence was higher in the calculous form (although without statistical significance). Two anaerobic infections with *Peptostreptococcus* and four *Candida* infections were noted, two being associated with other bacterial strains (Table 4). Seven cases of acalculous and sixty-nine calculous acute cholecystitis had more than one microbe isolated in cultures.

To determine if the severity of the acute cholecystitis influenced the proportion of positive bile cultures and the type of bacterial strain detected in acalculous and calculous acute cholecystitis, we performed a statistical analysis by adjusting microbiological data obtained by age (above versus below 60 years), perforation, Tokyo grade, acute kidney injury, and open versus laparoscopic surgery. The proportion of positive bile cultures was similar in acalculous and calculous acute cholecystitis (61.2% vs. 68%, *p* = 0.228). No difference regarding the proportion of isolated bacterial strains was found for *E. coli* (52.2% vs. 56.5%, *p* = 0.558), *Klebsiella* (26% vs. 34%, *p* = 0.204), *Enterobacter* (8.5% vs. 11.1%, *p* = 0.532), and *Streptococcus* (1.7% vs. 7%, *p* = 0.098); however, a much lower proportion of *Enterococcus* (26.2% vs. 4.9%, *p* < 0.001) and *Staphylococcus aureus* (1.7% vs. 11.7%, *p* = 0.013) was found.

Prophylactic antibiotherapy was prescribed in 261 of 272 cases with a positive bile culture (96%), in 138 of 143 sterile bile cultures (96.5%), and in 423 of 441 cases with no bile culture; in patients with a positive bile culture, the antibiotherapy was adequate (assessed by in vitro susceptibility) in 203 cases (77.8%) and inadequate in 58 cases; in 40 cases with inadequate empiric antibiotherapy, treatment was adjusted by antibiogram results, whereas in the remaining 18 cases, the evolution was favorable or patients were already discharged before antibiogram results arrived. Blood cultures were performed in 20 patients (with 8 positive cases); peritoneal fluid culture was performed in 19 patients (14 cultures being positive). Unadjusted mortality was similar in patients with a positive and sterile bile culture (6.3% each) and in patients with adequate (6.9%) or inadequate antibiotherapy (5.2%, *p* = 0.640). Adjusted mortality (by age, Tokyo grade, perforation, acute kidney injury, and open or laparoscopic surgery) was similar (4.8 vs. 6.8%, *p* = 0.401).

The microbiological spectrum of acalculous and calculous cholecystitis may be impacted in our study by the exclusion of acalculous cholecystitis cases associated with systemic infections. A sensitivity analysis was performed in patient subgroups stratified by severity (Tokyo grade II and III). The percentage of positive bile cultures was similar in Tokyo III compared to Tokyo II acute cholecystitis (32.1% vs. 34.4%, *p* = 0.809). The proportion of Gram-negative and Gram-positive bacteria was also similar (73.1% and 26.9% vs. 74.8% and 23.1%, *p* = 0.666); the presence of *Acinetobacter*, *Citrobacter*, *Serratia*, *Streptococcus*, and *Candida* was, however, noted only in Tokyo II grade acute cholecystitis. The Tokyo III subgroup had a higher proportion of patients aged 60 years or older (88.1 vs. 66.6, *p* = 0.004) and more frequent open surgery and acute kidney injury (73.8 vs. 37.6, *p* < 0.001, and 47.7 vs. 9%, *p* < 0.001); in-hospital mortality rate was also much higher in Tokyo III compared to Tokyo II patients (45.2 vs. 3.3%, *p* < 0.001). No differences regarding the rate of perforation, gangrene, and comorbidities were recorded.

**Figure 2:**
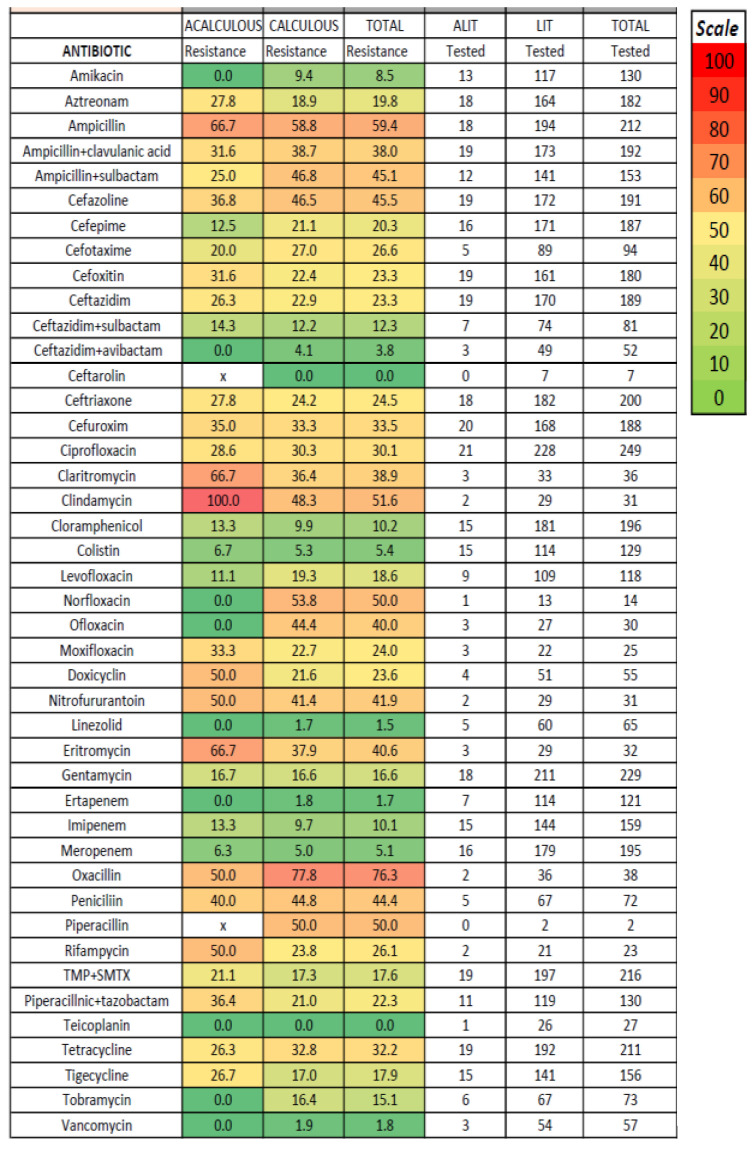
In vitro resistance to antibiotics for all patients with acute cholecystitis. x = in acute acalculous cholecystitis, piperacillin was not tested.

**Figure 3:**
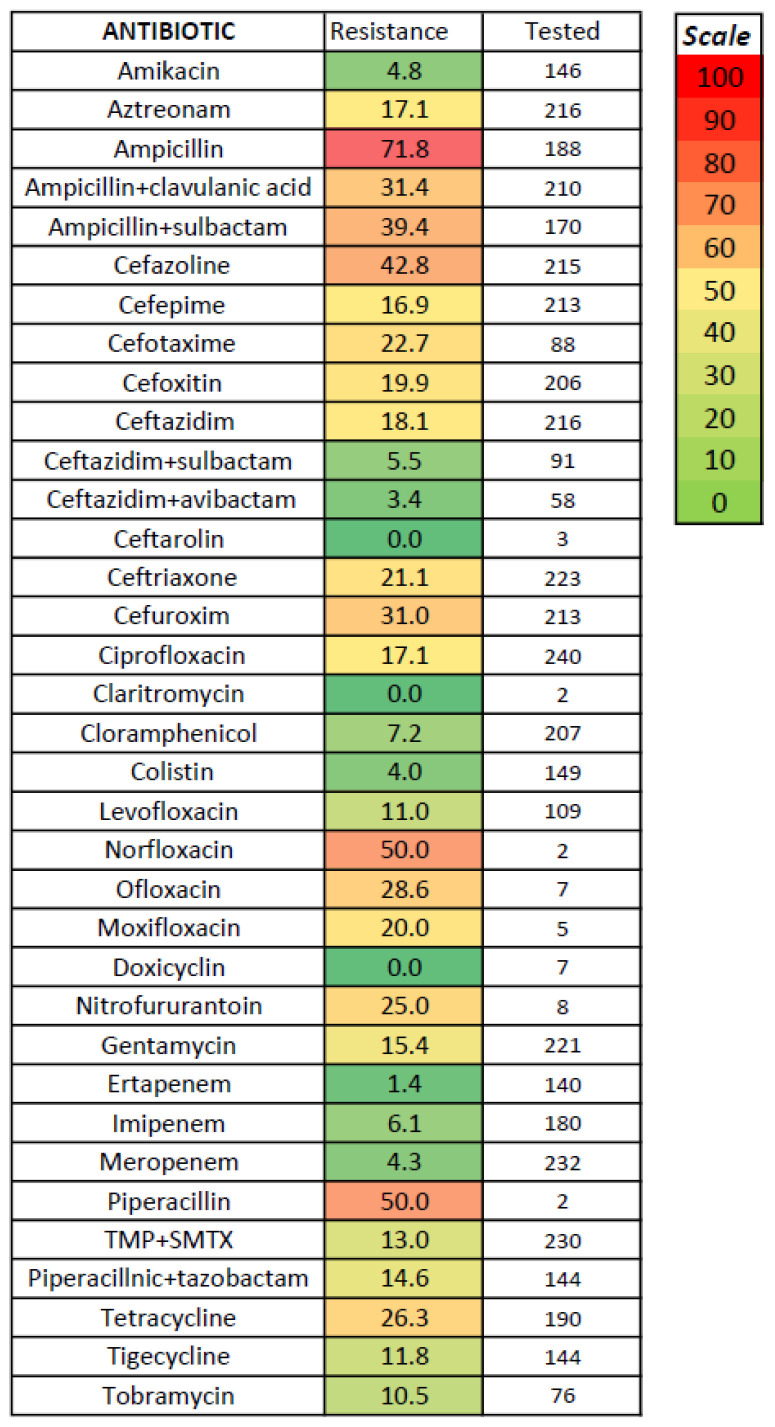
In vitro resistance to antibiotics for Gram-negative bacteria in acute cholecystitis.

**Figure 4:**
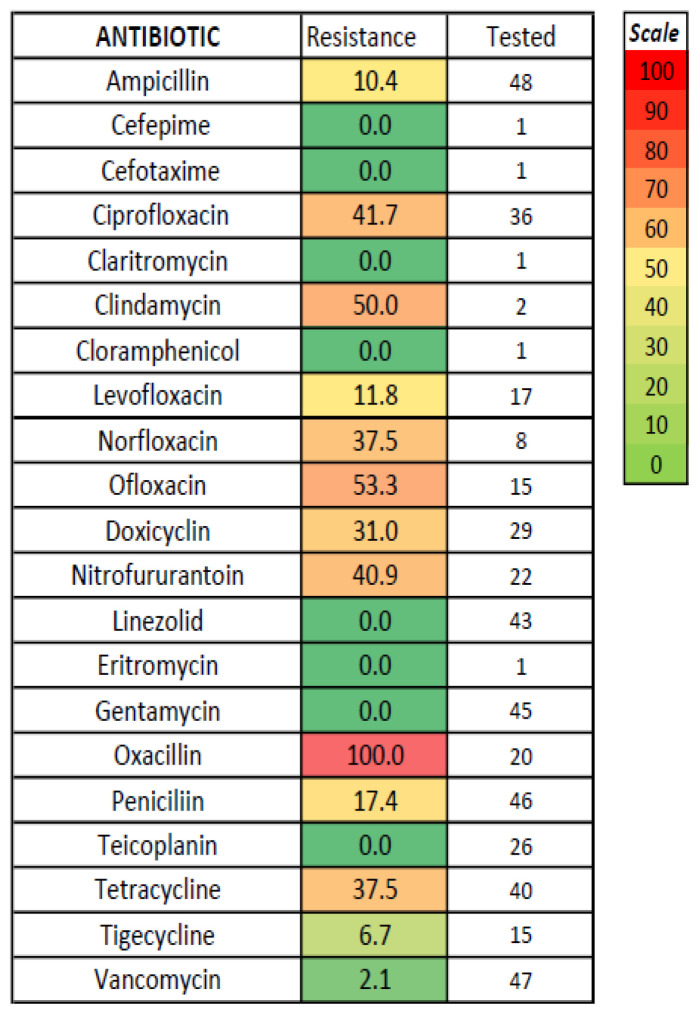
In vitro resistance to antibiotics for *Enterococcus* in acute cholecystitis.

**Figure 5:**
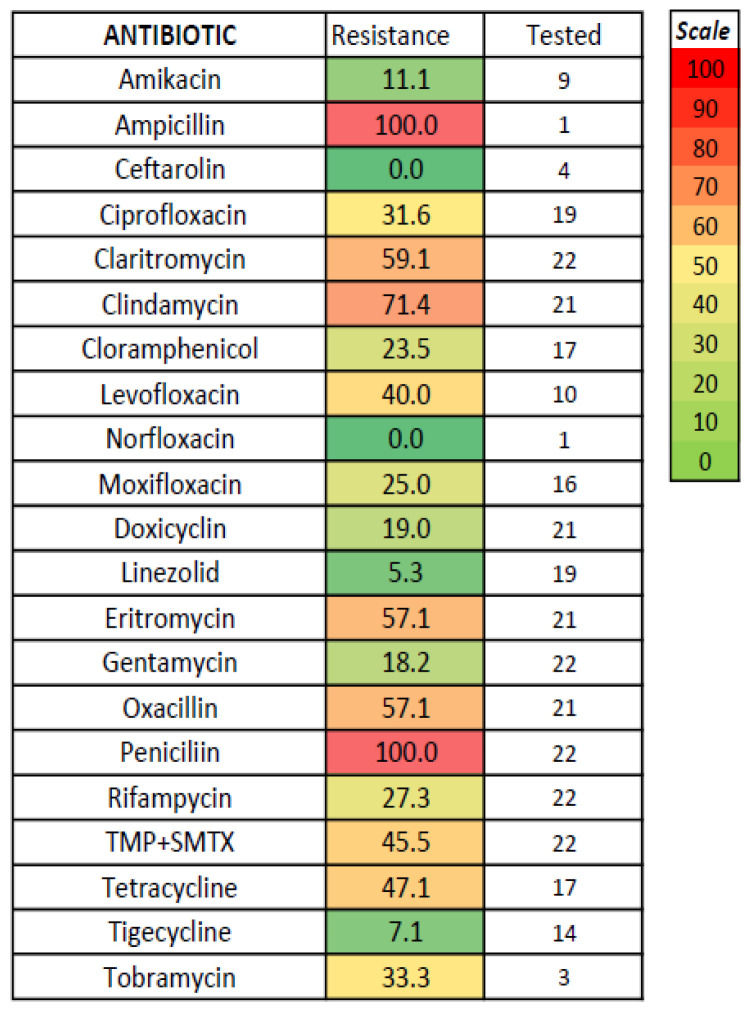
In vitro resistance to antibiotics for *Staphylococcus aureus* in acute cholecystitis.

No significant differences regarding antibiotic susceptibility testing were found between acute acalculous and calculous cholecystitis (Figure 2); the lowest resistance was noted for amikacin, carbapenems, chloramphenicol, and colistin (tested only for Gram-negative bacteria) and for vancomycin (tested only for *Enterococcus*)—Figure 3, Figure 4 and Figure 5. The combination of ceftazidime with either sulbactam or avibactam was also highly effective in vitro, although tested in only one-third of cases.

A total of 10 cases of ESBL-producing *E. coli* and *Klebsiella* strains were noted; 11 of the 19 *Staphylococcus aureus* infections were produced by MRSA strains. One VRE strain was recorded from the forty-eight infections. CPE presence was recorded in 21 of 268 strains, representing 7.8% (Table 5).

In the univariate analysis, in-hospital mortality was greater in patients aged 60 years or older (OR 55.489, *p* = 0.005), with Tokyo II and III grades (OR 4.072, *p* = 0.026, and OR 91.420, *p* < 0.001), open surgery (OR 21.670, *p* < 0.001), acute kidney injury (OR = 15.220, *p* < 0.001), acalculous acute cholecystitis (OR 6.058, *p* < 0.001), and perforation (OR 2.607, *p* = 0.005). In the multivariate analysis, age, Tokyo III grade, open cholecystectomy, and acute kidney injury were independent predictors for mortality—Table 6.

## 4. Discussion

In our study, acalculous acute cholecystitis represents 6.3% of total cholecystectomies, in line with the literature [1,2,3,4,5,6,7,8,9]. The main risk factors in our study were diabetes (OR = 2.049, 95% CI 1.1426 to 3.6730, *p* = 0.014), age (66.6 ± 13.2 vs. 61.4 ± 15.6, *p* = 0.014), and male gender (OR = 1.767, 95% CI 1.0123 to 3.0853, *p* = 0.043). No statistically significant association has been found with obesity, hypertension, or other cardiovascular disease; a higher frequency of previous stroke was noted, but it was outside statistical significance (*p* = 0.053). The association with cerebrovascular disease in the literature is unclear [7]. In several studies, acute acalculous cholecystitis was predominant in males [5,6,14], older patients [6,14], and patients with significant cardiovascular diseases, such as acute or chronic heart failure, acute myocardial ischemia, chronic coronary disease, aortic dissection, or hypertension [7,14]. In some studies, a younger age and the absence of chronic severe illness were noted [5]. Some of these diseases may induce AAC by acute hypoperfusion, whereas in other cases, microangiopathy or a vagally mediated reflex mechanism has been postulated [5,7,14]. In cerebrovascular disease, fasting, atherosclerosis, and higher bile concentration were proposed as mechanisms [7].

Although traditionally considered a disease encountered in severe illness, recent studies have shown that 77 to 90% of acute acalculous cholecystitis cases occur in outpatients with no critical illness present [5]. We found no statistical association with the presence of hypertension, although approximately 50% of patients with both acalculous and calculous acute cholecystitis had hypertension. An association with hypertension has been found in some studies [5,14].

No significant association with systemic viral or bacterial infection was found in our study; this association is frequently encountered in children [2] but is less likely in adults. Another explanation may be the inclusion of surgically treated acute acalculous cholecystitis, so cases that appeared during systemic infection or in critically ill patients and treated exclusively conservatively were skipped; forms that occur during an infectious disease are admitted in another dedicated hospital (Infectious Disease Hospital of Craiova) and were therefore excluded from our study. No statistically significant association has been found with COVID-19 infection (one acalculous and nine calculous acute cholecystitis cases); vasculitis and extensive thrombosis are postulated as the main mechanisms in cases associated with COVID-19 [9,16,17].

In our study, the mortality in acute acalculous cholecystitis was significantly higher than in calculous cholecystitis (18.5 vs. 3.6%, OR = 6.058, 95%CI 2.776–13.220, *p* < 0.001), had a longer hospitalization (mean 9.7 days vs. 8.4 days), and had an increased frequency of perforation (33.3 vs. 19.3%, OR = 2.087, 95%CI 1.154–3.774, *p* = 0.013) and phlegmonous forms (29.6 vs. 15.8%, OR = 2.238, 95%CI 1.211–4.136, *p* = 0.01); a higher proportion of acute acalculous cholecystitis cases had higher Dindo–Clavien grades. The higher mortality in acute acalculous cholecystitis was also emphasized in most studies [3], mainly as a result of more severe comorbidities; however, in other studies, the mortality was similar, especially in outpatient cases with no severe illness [5]. From a surgical perspective, mortality may be influenced by delays in establishing the diagnosis and, consequently, in proceeding to operative intervention. The absence of gallstones and the overlap of ultrasonographic criteria for acute cholecystitis (such as a thickened, double-layered gallbladder wall) with gallbladder alterations secondary to certain comorbid conditions (e.g., advanced hepatic cirrhosis or severe-stage heart failure) were factors that may postpone diagnostic confirmation.

In our study, the positivity of bile cultures was 60.5% and 66.2% in the two forms of acute cholecystitis, with no significant difference between acalculous and calculous cholecystitis; this was similar to the literature data, where 41–63% of bile cultures are positive [27], although values as low as 25% were noted [27,28,29]. The etiology was dominated by Gram-negative bacteria (mainly *E. coli* and *Klebsiella*), followed by *Enterococcus* and *Staphylococcus aureus* (with a higher percentage of the latter two bacteria noted in acalculous acute cholecystitis, although statistical significance was not attained). In several published studies, the most frequent isolated bacteria were *E. coli* and *Klebsiella*, followed by *Enterococcus*, *Staphylococcus*, *Enterobacter*, and *Citrobacter* [27,30,31,32,33,34,35]. In a large study including 6433 patients with acute cholangitis and cholecystitis, approximately 40% were *E. coli*, followed by *Klebsiella*, *Enterococcus*, and *Enterobacter*, with no significant differences between Tokyo I, II, and III grades [34]; another study of 217 patients with acute cholecystitis has found bile culture positivity rates to be proportional with Tokyo grade (Grade I 19.9%, Grade II 33.3%, and Grade III 70%) [33]. An increasing trend for Gram-negative bacteria and a decreasing prevalence of *Enterococcus spp* was also recorded [36]. Data regarding acalculous acute cholecystitis are very scarce; a study published in 2024 has found 195 cases of acute acalculous cholecystitis, with 60% of bile cultures being positive; the most frequent isolated bacteria were *E. coli* (22.6%) and *Klebsiella* (21%), followed by *Enterococcus* (7.2%), *Clostridioides* (6.7%), and *Enterobacter* (6.2%), but no susceptibility data were provided [37]. In another study, 3 of 11 patients with acute acalculous cholecystitis had positive bile cultures (2 with *Klebsiella spp.* and 1 with *E. coli*) [5].

The importance of the type of bile infection and antibiotic susceptibility in acute cholecystitis represents a complex issue. Since most acute cholecystitis cases will require cholecystectomy (sometimes in emergency settings), preoperative and postoperative antibiotherapy are useful to reduce the risks of bloodstream infections, septic shock, and infective postoperative complications [38,39,40], and inadequate antibiotherapy represents a risk factor for poor outcome [30,41]; an accurate surgical intervention with removal of the gallbladder as the source of infection and proper peritoneal drainage are of utmost importance to control the infection [42]. However, some studies in the literature have shown that a positive bile culture did not influence mortality and evolution [43,44]. When surgery is riskier or contraindicated because of significant comorbidities (sometimes more frequent in acute acalculous cholecystitis) and conservative treatment with or without percutaneous cholecystostomy is the first choice, the knowledge of etiology and antibiotic susceptibility is important for choosing the antibiotherapy. Since increasing resistance to antibiotics has been noted recently for various types of bacterial infections and in some cases, a bacterial shift toward more frequent Gram-positive infections has occurred [45], a study detailing the etiology and susceptibility is appropriate. In a study detailing the microbiological pattern in acute calculous cholecystitis, 37% of patients received improper antibiotherapy, judged by in vitro susceptibility [46]; in another study, a 67.6% concordance between empiric treatment coverage and culture isolate susceptibility was noted [47]. Geographical differences regarding both issues, as well as the differences between the pathogenesis of acalculous and calculous acute cholecystitis (with an ischemic mechanism being more important), may further emphasize the importance of studies in acute acalculous forms.

Our study confirms a concerning trend of increasing antibiotic resistance in acute cholecystitis, similar to other types of infections. The prevalence of antibiotic-resistant bacteria was significant, with 61.1% MRSA strains, 7.8% CPE (with higher proportions for *Acinetobacter*, *Pseudomonas*, and *Citrobacter*), 3.7% ESBL strains, but only one VRE strain. The resistance to cephalosporins was significant, with up to 35% of cases, and even the fourth-generation cephalosporin (Cefepime) was associated with a resistance rate reaching one-fifth of total cases; these data were similar to other studies [28,36]. We had insufficient data regarding the susceptibility to fifth-generation cephalosporins such as ceftaroline, which was tested for only seven isolated bacterial strains; ceftaroline is mostly efficient against Gram-positive bacteria, but in our patient group, all four MRSA tested strains and three Gram-negative bacteria (two *E. coli* and one *Klebsiella* strain) were susceptible in vitro. The resistance to quinolones was similar or even higher. In Gram-negative infections, Amikacin, some combinations of cephalosporins with sulbactam or avibactam, and carbapenems seem efficient in vitro. In *Enterococcus* and *Staphylococcus spp* infections, linezolid and tigecycline were efficient in vitro, and only 1 of 47 strains of *Enterococcus* tested for Vancomycin was resistant (no *Staphylococcus* strain was tested for Vancomycin); the appearance of vancomycin-resistant *Enterococci* was noted in 16.2% in a study [31]. Our obtained data were similar to other studies [28,31], with less than 5% in vitro resistance against carbapenem, beta-lactam antibiotics, glycopeptide antibiotics, and linezolid [28]. In a study of 147 culture-positive cases of 262 acute cholecystitis, cefotetan (a second-generation cephalosporin) was more effective (less than 5% resistance rate) against *E. coli* and *Klebsiella* compared to cefotaxime (30.2% resistance rate), whereas ceftazidime was the most effective cephalosporin antibiotic against *Enterobacter* (77.8%); ciprofloxacin was effective against *Klebsiella* and *Enterobacter*, but only 60.4% of E. coli were susceptible [31]. In a study of 54 patients with culture-positive acute cholecystitis and 83 bacterial strains detected; the resistance rate was 35.6% for cefazolin, 24.4% for cefoxitin, 19.4% for ceftazidime, 15.4% for cefotaxime, 13.7% for cefepime, 23% for ciprofloxacin, 36.4% for levofloxacin, and 18.9% for piperacillin–tazobactam, while the resistance rate for carbapenems was low [28]. No particular resistance was noted in our study in *Streptococcus* or anaerobic infection. We found no recent studies regarding the microbiologic spectrum or susceptibility in acute cholecystitis in Romania; a study in Croatia and another in Turkey found similar data regarding microbiological profile and antibiotic resistance [30,40].

The association of two or more bacterial strains can further complicate the resistance issue, since it may require an extensive association of antibiotics. In Gram-negative acute cholecystitis, current guidelines recommend amoxicillin with clavulanic acid and third- or fourth-generation cephalosporins (with or without metronidazole) as the first choice, with fluoroquinolones, piperacillin–tazobactam, and carbapenems as the second option, guided by the severity of the cholecystitis and disease duration [36,40]. In our study, an empiric association between a carbapenem and either vancomycin, linezolid, or amikacin may fit all bacterial etiologies (based on the susceptibility in vitro). This therapeutic regimen may be, however, useful only in severe cases complicated with septic shock, or in cases managed conservatively because of severe comorbidities, and therapy adjustment after the antibiogram may permit targeted antibiotherapy when bile cultures arrive after cholecystectomy (not available in cases managed conservatively), in positive bile cultures after cholecystostomy, and in cases with a positive blood culture. In patients with community-acquired infection, early surgery and low risk for MDR bacteria, amoxicillin–clavulanic acid combination, or ceftriaxone/cefotaxime plus metronidazole is currently indicated, whereas in cases with late surgery, patients that are immunocompromised, or cases with healthcare-associated infection, a piperacillin–tazobactam combination or a carbapenem such as ertapenem is currently indicated [31]. It is also important to point out that the association of two or more antibiotics, although potentially recommended by in vitro susceptibility, may not translate into a clinical benefit compared to a single antibiotic regimen [40,48].

The occurrence of *Candida* cholecystitis represents a rare event, usually associated with immunosuppression, diabetes, and malignancy; acalculous necrotizing *Candida* cholecystitis has also been described [28,49,50]. In a study, 1% of bile cultures were positive for *Candida* [46], and in another, 2.4% of cholecystectomies [51]. In our study, four cases of acute cholecystitis with *Candida* albicans isolates were noted (all being calculous cholecystitis); in two cases, *Candida* was the only strain found in culture. The evolution was unremarkable and without complications. No association with diabetes, malignancy, or other immunosuppressive status was noted; one case was recorded in a patient with liver cirrhosis and another in a patient with chronic obstructive pulmonary disease.

Several limitations of the study were noted. The retrospective and unicentric design of our study has limited the generalizability of our findings, although the local particularities of the microbiological pattern and antibiotic resistance are very important for adapting current guidelines to local data. Non-surgical cases of acute acalculous cholecystitis were excluded by study design, and cases associated with systemic infections were therefore not included in our data, which may influence the evaluation of the etiology of acute acalculous cholecystitis. Antimicrobial susceptibility testing was influenced by the local availability of the testing kits, and some antibiotics were not tested in all samples. The impact of in vitro resistance on mortality or postoperative complications may be difficult to estimate, because in patients with acute cholecystitis, antibiotherapy did not represent the definitive treatment, and decreasing the concentration of local and systemic bacteria may be enough to control mortality and complications in cases of prompt and proper surgery. Future studies, including multicentric protocols, more extensive and detailed epidemiological data, inflammatory markers such as C-reactive protein, NLR, or pro-inflammatory cytokines, and also in-depth exploration of the precise relationship between preoperative antibiotic treatment and mortality or postoperative complications, are warranted. Additionally, studies employing more accurate tests for detecting microorganisms—such as next-generation sequencing—could be useful in better characterizing bile colonization in acute cholecystitis, since a significant number of microorganisms are not identified by bile culture [52].

## 5. Conclusions

Acalculous acute cholecystitis was rarely encountered in cholecystectomy specimens, mainly in older males with diabetes; higher rates of complications and in-hospital mortality were recorded. Bile cultures were positive in 60.5 to 66.2% of cases, predominantly with Gram-negative, *Enterococcus*, and *Staphylococcus* species; similar isolated strains were noted in bile cultures from acalculous and calculous acute cholecystitis in our study. Significant in vitro resistance to cephalosporins and quinolones was noted.

## Figures and Tables

**Table 1:** Tokyo classification of acute cholecystitis [24].

Grade I (mild)	Acute cholecystitis with no criteria for grade II or III
Grade II (moderate)	Any of the following:
	-white blood cells >18,000/mm^3^
	-palpable soft mass in the right hypochondrium
	-symptoms for more than 72 h
	-marked local inflammation (gangrenous or emphysematous cholecystitis, peri-cholecystic or hepatic abscess, choleperitoneum)
Grade III (severe)	Associated with organ/system dysfunction:
	-hypotension requiring at least 5 µg/min dopamine or any epinephrine dose -decreased level of consciousness
	-PaO_2_/FiO_2_ < 300
	-oliguria or creatinine level > 2 mg/dL
	-INR > 1.5
	-platelet count < 100,000/mm^3^

**Table 2:** Dindo–Clavien classification of cholecystectomy [24].

Grade I	Any deviation from the normal intraoperative or postoperative course, including the need for pharmacologic treatment other than antiemetics, antipyretics, analgesics, diuretics, electrolytes, or physiotherapy
Grade II	Complications needing only the use of intravenous medications, total intravenous nutrition, or blood transfusion
Grade III	Complications needing surgical, endoscopic, or radiologic intervention under local or general anesthesia
Grade IV	Life-threatening complications requiring ICU management—single or multiple organ dysfunction (including hemodialysis)
Grade V	Death

**Table 3:** Characteristics of patients with acute acalculous and calculous cholecystitis.

	Acalculous Cholecystitis N = 54	Calculous Cholecystitis N = 802	*p*-Value
Age: yrs Mean ± Std (Min-Max)	66.6 ± 13.2 (19–88)	61.4 ± 15.6 (18–96)	0.014
Gender M/F (No, % of females)	31/23 (42.6)	455/347 (56.7)	0.043
Tokyo classification			0.005
-I	14 (25.9)	321 (40.0)	
-II	33 (61.1)	446 (55.6)	
-III	7 (13.0)	35 (4.4)	
Complications (%)			
-gangrenous	27 (50)	318 (39.7)	0.136
-phlegmonous	16 (29.6)	127 (15.8)	0.010
-perforation	18 (33.3)	155 (19.3)	0.013
-fistula	1 (1.9)	12 (1.5)	0.836
Comorbidities (%)			
-obesity	13 (24.1)	235 (29.3)	0.412
-hypertension	29 (53.7)	416 (51.9)	0.794
-cardiovascular diseases	35 (64.8)	449 (56.0)	0.205
-respiratory diseases	5 (5.1)	41 (9.3)	0.191
-liver cirrhosis	1 (1.9)	11 (1.4)	0.771
-peptic ulcer	1 (1.9)	5 (0.6)	0.295
-diabetes	19 (35.2)	168 (20.9)	0.014
-renal diseases	1 (1.9)	19 (2.4)	0.808
-neurological diseases	6 (11.1)	40 (5)	0.053
-tumors	1 (1.9)	17 (2.1)	0.894
-COVID-19 infection	1 (1.9)	9 (1.1)	0.629
-*Clostridium difficile* infection	2 (3.7)	6 (0.7)	0.029
-rheumatological diseases	0 (0)	3 (0.4)	0.653
Laboratory			
-Hb (g/dL)	12.3 ± 1.8	13 ± 1.8	0.002
-leucocyte count	13,756 ± 6571	13,079 ± 6498	0.562
-neutrophil count	11,630 ± 6261	10,228 ± 6302	0.083
-lymphocyte count	1165 ± 662	1701 ± 898	<0.001
-platelet count	226,826 ± 92,327	256,164 ± 93,060	0.025
-urea (mg/dL)	75 ± 61	44 ± 25	<0.001
-creatinine (mg/dL)	1.59 ± 1.65	1.01 ± 0.74	<0.001
-serum glucose (mg/dL)	134 ± 66	120 ± 56	0.129
-ALT (IU/L)	110 ± 145	68 ± 118	<0.001
-AST (IU/L)	109 ± 137	57 ± 99	<0.001
-serum amylase (IU/L)	103 ± 167	87 ± 244	0.482
-total bilirubin (mg/dL))	2.25 ± 2.82	1.31 ± 1.80	0.014
-direct bilirubin (mg/dL)	1.37 ± 2.08	0.70 ± 1.36	0.001
Charlson comorbidity index (mean ± standard deviation)	3.3 ± 1.9	2.5 ± 2	0.002
Surgery type			<0.001
-laparoscopic	23 (42.6)	563 (70.2)	
-open	26 (48.1)	187 (23.3)	
-conversion	5 (9.3)	52 (6.5)	
Complications (Dindo–Clavien grade)			<0.001
-0	39 (72.2)	746 (93)	
-1/2	2 (3.7)	12 (1.5)	
-3/4	3 (5.6)	15 (1.8)	
Mean hospital stay (days)	9.7 ± 5.6	8.4 ± 5.1	<0.001
In-hospital mortality (%)	10 (18.5)	29 (3.6)	<0.001

**Table 4:** The microbiological spectrum in acalculous and calculous acute cholecystitis.

	Acalculous Cholecystitis N = 54	Calculous Cholecystitis N = 802	*p*-Value
Positive patients	23 (60.5)	249 (66.2)	0.3660
Positive cultures	33 (67.3)	327 (71.7)	0.5279
Gram-negative	28 (84.9)	242 (74)	0.1775
*-E. coli*	14 (42.4)	117 (35.8)	0.4992
*-Klebsiella*	9 (27.3)	65 (19.9)	0.2020
*-Enterobacter*	3 (9.1)	19 (5.8)	0.4759
*-Serratia*	2 (6.1)	3 (0.9)	0.0391
*-Pseudomonas*	0 (0)	13 (4)	0.4587
*-Proteus*	0 (0)	7 (2.1)	0.7502
*-Citrobacter*	0 (0)	10 (3.1)	0.5766
*-Acinetobacter*	0 (0)	8 (2.4)	0.5505
Gram-positive	5 (15.1)	79 (24.2)	0.9862
*-Enterococcus*	2 (6.1)	46 (14.1)	0.2020
*-Staphylococcus aureus*	1 (3)	18 (5.5)	0.5384
*-Staphylococcus coagulase-negative*	1 (3)	3 (0.9)	0.3058
*-Streptococcus*	1 (3)	12 (3.7)	0.8372
Anaerobes			
-*Peptostreptococcus*	0 (0)	2 (0.6)	0.6789
Candida spp.	0 (0)	4 (1.2)	0.9725

**Table 5:** The prevalence of MDR, XDR, ESBL, KPC, MRSA, and VRE in acalculous and calculous acute cholecystitis.

	MDR (%)	XDR (%)	ESBL (%)	CPE (%)	MRSA (%)	VRE (%)
All bacterial strains	155/354 (43.8)	6/354 (1.7)	-	-	-	-
Gram-negative			10/268 (3.7)	21/268 (7.8)	-	-
*-E. coli*	55/131 (42)	0/131 (0)	3/131 (2.3)	5/131 (4.1)	-	-
*-Klebsiella*	40/74 (54.1)	1/74 (1.4)	6/74 (8.1)	6/74 (8.1)	-	-
*-Enterobacter*	14/22 (63.6)	0/22 (0)	1/22 (4.5)	0/22 (0)	-	-
*-Serratia*	3/11 (27.3)	1/11 (9.1)	0/11 (0)	1/11 (9.1)	-	-
*-Pseudomonas*	4/10 (40)	0/10 (0)	0/10 (0)	4/10 (30.8)	-	-
*-Proteus*	5/8 (62.5)	3/8 (37.5)	0/8 (0)	0/8 (0)	-	-
*-Citrobacter*	6/7 (85.7)	0/7 (0)	0/7 (0)	2/7 (28.6)	-	-
*-Acinetobacter*	5/5 (100)	0/5 (0)	0/5 (0)	3/5 (60)	-	-
Gram-positive						
*-Enterococcus*	5/48 (10.4)	0/48 (0)	-	-	-	1/48 (2)
*-Staphylococcus aureus*	13/18 (72.2)	1/18 (5.6)	-	-	11/18 (61.1)	-
*-Staphylococcus coagulase-negative*	4/5 (80)	0/5 (0)	-	-	0/5 (0)	-
*-Streptococcus*	0/13 (0)	0/13 (0)	-	-	-	-
Anaerobes			-	-	-	-
-*Peptostreptococcus*	1/2 (50)	0/2 (0)	-	-	-	-

**Table 6:** Risk factors for in-hospital mortality in acute cholecystitis.

Factor	Univariate Analysis Odds Ratio (95% CI), *p*	Multivariate Analysis Odds Ratio (95% CI), *p*
Age ≥60/<60 years	55.489 (3.399–906), 0.005	69.392 (4.268–1128.209), 0.003 (0.24 to 25.7)
Tokyo grade		
-Grade III vs. I	91.420 (25.192–331.753), <0.001	5.126 (1.835–14.325), 0.002
-Grade II vs. I	4.072 (1.183–14.008), 0.026	3.609 (0.687–6.320), 0.067
Renal injury	15.220 (7.616–30.414), <0.001	4.974 (2.335–10.597), <0.001
Acalculous cholecystitis	6.058 (2.776–13.220), <0.001	2.164 (0.961–4.872), 0.062
Open cholecystectomy	21.670 (7.617 to 61.649), <0.001	7.807 (3.365–18.112), <0.001
Perforation	2.607 (1.336 to 5.085), 0.005	1.920 (0.971–3.799), 0.061

## Data Availability

The datasets obtained and used during the current study are available upon request from the corresponding author.

## Abbreviations

The following abbreviations are used in this manuscript:

AAC	Acalculous acute cholecystitis
AIDS	Directory of open access journals
ALT	Alanine aminotransferase
AST	Aspartate aminotransferase
ERCP	Endoscopic retrograde cholangiopancreatography
ESBL	Extended-spectrum beta-lactamase
EUS	Endoscopic ultrasound
ICU	Intensive care unit
MDR	Multidrug-resistant
MRSA	Methicillin-resistant *Staphylococcus aureus*
OR	Odds ratio
XDR	Extensively drug-resistant
VRE	Vancomycin-resistant *Enterococci*
